# Bibliography

**Published:** 1898-02

**Authors:** 


### Bibliography.

A Quiz Manual of Histology, General and Dental. By Charles B. Reed, M.D., Professor of General Histology, Northwestern University Dental School, etc., and Frederick B. Noyes, B.A., D.D.S., Professor of Dental Histology (same school). Chicago: The W. T. Keener Co., 1897.

This is a thin volume of 203 pages, consisting, as its name implies, of questions and answers. The authors believe that in "an experience of some years in teaching histology . . . that such a manual as the present would be of value in giving him [the student] a better foundation for his subsequent study."

Criticism of the general contents of the book is not possible, for the questions and answers are framed each to suit the other, and are, of course, based on accepted knowledge. It is doubtful whether the average student would care to make a work of this kind a basis for study. Histological work tells its own story as the student proceeds in his investigations, and all important points are firmly fixed in his mind as he advances step by step with his microscope and reagents.

The most important use of such a book will be to those who study histology through a memorizing of facts, and as this is equivalent to no knowledge, it is impossible to see how this work can be of value to students. To the quiz teacher and to boards of examiners it would be invaluable, and for their use it is recommended without any reservations.

An Epitome of the History of Medicine. By Roswell Park, A.M., M.D., Professor of Surgery in the Medical Department of the University of Buffalo, etc. Illustrated with Portraits and other Engravings. Philadelphia, New York, Chicago: F. A. Davis Company, publishers, 1897.

The history of professions is of profound interest, even to the laity, and this is especially true of medicine, for it means the history of the advances men have made from darkness to light, and the oftentimes ineffectual efforts to master the ills of humanity. The history of medicine, therefore, is practically the history of civilization, and, as the present century has witnessed the greatest advances in all directions of mental effort, it is not surprising that its last half should have shown greater advances than at any equal period in its history.

The work of Professor Park would naturally be of value to medical men; but as the story is told in a style at once attractive and instructive, the reader is lured on from the first page to the last.

The author truly states in his preface that "the history of medicine is really a history of human error and of human discovery. During the past two thousand years it is hard to say which has prevailed. Notwithstanding, had it not been for the latter, the total of the former would have been vastly greater. A large part of my effort has been devoted to considering the causes which conspired to prevent the more rapid development of our art. If among these the frowning or forbidding attitude of the church figures most prominently, it must not be regarded as any expression of a quarrel with the church of to-day."

This history is largely made up from the characters that figured in medicine in various ages, differing in this respect from the "History of Medicine" by Robley Dunglison, M.D., which dwelt largely upon the various schools of medicine, relegating the exponents thereof to a secondary position. While this is true of the book as a whole, there is so much of interesting matter interwoven in its pages with these biographical sketches that the actual changes and the motives that induced these in the various periods are vividly portrayed.

In writing of Ambroise Paré, the author quotes this from his work on surgery: "For my part, I have dispensed liberally to everybody the gifts that God has conferred upon me, and I am none the worse for it, just as the light of a candle will not diminish, no matter how many may come to light their torches by it." If all connected with medicine and its specialties would adopt this noble sentiment, the professional spirit would be more in fact and less in name than at present.

The author's prejudices lead at times to narrowness of thought. Especially is this marked where he discusses the work of Gall and Spurzheim. "They thus founded the pseudo-science denominated phrenology, which we now know has practically nothing to justify itself." This, in all probability, settles the question in the author's mind, but will not in those who have intelligently examined the subject.

The book is enriched with a profusion of engravings taken from old paintings and portraits of distinguished men of all times in the medical profession, ending with Lord Lister, M.D. A very excellent picture of Professor Gross accompanies this collection.

The compilation of such a work is a labor of great magnitude, but as it has been, undoubtedly, a labor of love, the reward must come in that direction. It should, however, be in the hands of all specialists in medicine, and this includes dentists.

The author devotes his closing chapter to this specialty, and says, "There is no reason why there is not more excuse for true oral surgeons than there is for any other class of specialists, save possibly those who treat the eye." The dental profession will not think the exception well taken, for while the assistance rendered by the ophthalmologist is of immeasurable value, that of the stomatologist covers not only the oral cavity but the entire digestive tract; and if his work is properly performed, he may influence for good the entire organism.

This work of Professor Parks must be regarded as one of the most valuable for reference, as it is full of facts not readily obtained elsewhere.

About Children. Six lectures given to the Nurses in the Training School of the Cleveland General Hospital in February, 1896. By Samuel W. Kelley, M.D., Professor of Diseases of Children in the Cleveland College of Physicians and Surgeons (Medical Department Ohio Wesleyan University); Pædiatrist to the Cleveland General Hospital; Consulting Physician to the Cleveland City Hospital; President, 1896 and 1897, Ohio State Pædiatric Society; Editor *Cleveland Medical Gazette.* 180 pages. Price, in buckram, postpaid, $1.25, net. Cleveland: The Medical Gazette Publishing Company, 1897.

As the title indicates, these lectures were prepared for nurses, and this view disarms, in a great measure, adverse criticism, for a full consideration of the subjects could not be expected.

The diseases of children have received at all times the attention the subject deserved, and the valuable works published in the past attest the fact of their importance in medical practice and their value in its literature.

The author carries his hearers and readers through all the stages of infant life from the new-born to the final eruption of all the deciduous teeth, or between the second and third year. This period, though at times superficially treated, is, upon the whole, satisfactory. There are, however, very great lapses observable by the dental reader, which is not an unusual fact with medical writers, for they frequently seem possessed with the idea that all knowledge came to them by virtue of their special work, the care of the human body, and that the acquirement of information from a special branch would be a lowering of the dignity of the profession. Had our author consulted the standard works of dentistry he would not have made a chapter on dentition as weak as the one in this book. The following quotation will illustrate this: "The popular opinion is that a child ought to have diarrhœa while teething, but this and other disorders should be attended to just the same during teething as at any other period. . . . The upper teeth are likely to cause more disturbance than the lower. It is true that the cutting of the 'eye-teeth' (the canines) may through their nervous connection affect the eyes," etc.

The author delivered this series of lectures to nurses, and these should have been informed of the possible pathological complications attending dentition, but while a few of the sequelæ of dentition are mentioned, not one word as to the cause or simple remedy. Patience has ceased to be a virtue in this persistently setting aside, by medical men, of known facts. If it were simply a matter of prejudice it might be left severely alone, but as negligence or profound ignorance of the subject endangers the lives of thousands of infants, the neglect becomes intolerable, and amounts to a crime.

It is to be hoped, if the author ever gets out a second edition, he will consult recognized authorities on dentition. It will give added value to an otherwise excellent work.

